# Comparative analysis of compressible solver schemes for underexpanded jet aerodynamics with Schlieren validation

**DOI:** 10.1038/s41598-026-44651-w

**Published:** 2026-04-01

**Authors:** Sajda S. Alsaedi, Laith A. Al-Sadawi, Luttfi A. Al-Haddad, Amar S. Abdul-Zahra, Muath Odeh, Aymen Flah, Ahmed Ali Farhan Ogaili

**Affiliations:** 1 College of Mechanical Engineering, University of Technology- Iraq, Baghdad, Iraq; 2 Department of Basic Sciences, Middle East University, Amman, 11831 Jordan; 3 ENET Centre, CEET, VSB-Technical University of Ostrava, Ostrava, 708 00 Czech Republic; 4 Applied Science Research Center, Applied Science Private University, Amman, 11931 Jordan; 5 Center for Research Impact and Outcome, Chitkara University, Punjab University, Rajpura, Punjab 140401 India; 6 Department of Mechanical Engineering, Mustansiriyah University, Baghdad, Iraq

**Keywords:** Converging nozzle, Schlieren technique, Mach disk, Pressure-based solver, Density-based solver, Engineering, Mathematics and computing, Physics

## Abstract

The accurate prediction of compressible high-speed jet flows remains a critical challenge in computational aerodynamics due to the strong coupling between shock waves, expansion fans, shear-layer turbulence, and mixing processes downstream of nozzles. While density-based (DB) solvers are conventionally used for supersonic and transonic regimes, pressure-based (PB) solvers have recently gained attention for their reduced computational cost; however, their suitability for underexpanded jet modeling remains insufficiently explored. This study provides a systematic evaluation of PB and DB solvers available in ANSYS Fluent for simulating compressible flow discharged from an axisymmetric convergent nozzle over a range of nozzle pressure ratios (PR = 1.92–5). Experimental Schlieren flow visualization was conducted using a custom optical setup to qualitatively assess shock structures and validate near-field flow features. Numerical simulations employed compressible Reynolds Average Navier-Stokes (RANS) formulations with a standard k–ε turbulence model, structured quadrilateral meshes, and consistent boundary conditions for both solvers. Validation against published measurements demonstrated that both solvers accurately predict Mach disk formation and the streamwise location of the first shock cell, with a maximum deviation of 4.5% in peak Mach number. For PR > 1.92, both solvers captured the characteristic diamond shock pattern and the progressive increase in shock cell strength and spacing; the first shock cell occurred at X/De ≈ 1.09, 1.57, and 2 for PR = 3, 4, and 5, respectively. While PB and DB solvers exhibited comparable performance in resolving centerline Mach number and pressure oscillations, the DB solver overpredicted turbulent kinetic energy in the far-field subsonic region due to its known sensitivity at low Mach numbers. Discharge coefficients predicted by both solvers showed close agreement, with differences < 0.2%. Results demonstrate that the PB solver, despite being traditionally associated with incompressible and low-speed flows, can reliably model underexpanded supersonic jets at significantly reduced numerical cost. The findings provide practical guidance for CFD practitioners seeking cost-effective tools for compressible nozzle flow modeling and contribute to broader discussions on solver strategy selection for high-speed aerodynamic simulations.

## Introduction

High-speed compressible jets issuing from convergent and convergent–divergent nozzles play a critical role in propulsion and industrial gas discharge systems, where complex interactions between shock waves and mixing processes strongly influence performance and stability^1,2^. The prediction and control of these flow features remain central challenges in modern aerothermodynamics, as shock-induced separation, Mach disk formation, and shock–shear layer interactions govern thrust efficiency, noise generation, and thermal loading. Several experimental studies have been conducted to examine the flow structure downstream convergent and convergent -divergent nozzles. Due to its wide range of engineering applications such as propulsion in the aircraft industry, high-speed gases released from volcanoes during eruptions^3,4^, cold and hot underexpanded jets have been extensively studied both numerically and experimentally. However, significant problems still require further investigation, especially those related to understanding turbulent mixing regions and predictions of flow structure downstream the nozzle^5^. Based on the initial pressure ratio, total to atmospheric pressure ratio, the flow outside a converging nozzle can either be in subsonic or sonic regimes. When the discharged flow from the convergent nozzle has an exit pressure greater than the atmospheric pressure, an underexpanded jet may be released. In this case, the flow expands outside the nozzle with a series of expansion and compression shock waves. Depending on the pressure ratio, underexpanded converging nozzles can be either moderate or highly underexpanded^6^.

Underexpanded free jets that propagate from convergent or convergent divergent nozzles into quiescent air have been experimentally and numerically examined. For instance, with special attention paid to the prediction of the nearfield and far field flow zones, Franquet et al.^7^ performed a comprehensive review of the experimental investigations on axisymmetric underexpanded jets. Researchers have conducted several experimental studies using various techniques, including PIV, LDA, pitot tubes, and Schlieren flow visualization, to investigate the behavior of free underexpanded jets. For example, Trumper et al.^8^ conducted an experimental study to investigate the effect of the outlet condition on the subsonic and supersonic jet of a converging nozzle at different nozzle PR using LDA.

For the underexpanded jet stream, it was found that changing nozzle exit geometry results in a jet with a shorter potential core and a different shock structure. Zapryagaev et al.^9^ examined the effect of nozzle shape on the shock structure downstream region of the nozzle. André et al.^10^ found that Laser Doppler Anemometer LDA is the optimum method for capturing shock wave structures. Moreover, Henderson et al.^11^ used the Particle Image Velocimetry PIV system to investigate the Mach disc and the associated shear layer downstream an underxepanded convergent nozzle. The study concluded that the hypothesized recirculation that should follow the Mach disk did not exist. Seeking to understand the relation of the tone generated because of the impingement of the jet stream with a downstream plate^12^, found that the generated circulation zone between the jet and the plate does not affect the jet compared to the location of the plate and the strength of the first shock wave cell^13^. examined the effect of the interaction between the large-scale structures and the underexpanded hypersonic jets. Panda and Seasholtz^14^ conducted an experimental and numerical investigation of underexpanded free jets using the Rayleigh scattering technique. The result clearly showed the interaction between the vortex and shock cell structures due to the presence of pressure fluctuation on the boundary of the jet plume.

Although experimental visualization techniques such as Schlieren imaging^15^, shadowgraph^16^, Particle Image Velocimetry (PIV)^17,18^, and Laser Doppler Anemometry (LDA) have provided valuable insight into underexpanded jet behavior and shock cell topology^19,20^, the complexity and the need for expensive non-intrusive measuring devices, several numerical studies have been conducted to predict the devolvement of the supersonic jet plume in the vicinity of underexpanded converging nozzles^21–30^. For instance, Volkov^22^ used computational CFD code to investigate the effect of nozzle PR on the structure of an underexpanded jet and the properties of the flow downstream a converged nozzle. The study showed that the CFD solver could predict the jet plum downstream of the nozzle, and the results were coherent with previously conducted experimental measurements. Fukunaga et al.^23^ calculated the density of underexpanded microjets from a round convergent nozzle at various nozzle PRs. The flow topology of the near-field shock structures was illustrated successfully. In addition, Duronio et al.^24^ utilized the density-based solver within the OpenFOAM library to simulate underexpanded jets for methane injection. The results provided an insight into the structure of an underexpanded jet, shocks, flow field generation, and evolution of mixing activity outside the jet. A novel design of an asymmetrical convergent nozzle was successfully evaluated using the commercial CFD tool ANSYS (CFX)^26^. The study revealed that the new design has a significant effect on the fluid flow inside the nozzle. The performance of the rhoCentralFoam compressible solver that is available in OpenFOAM opensource software was evaluated in modeling an underexpanded round jet^27^. The study exhibited an error of 4% between the predicted and the experimentally calculated length of the first shock cell. In addition, the used code showed better performance than the commercial CFD software. A home-built Reynolds-Averaged Navier–Stokes RANS code was utilized to investigate supersonic underexpanded jet discharged into ambient atmospheric pressure^28^. It was found that as the nozzle pressure ratio PR increases, the distance at which the first shock occurs, and the diameter of the jet increases.

The CFD design optimization and performance prediction across a broad spectrum of pressure ratios and nozzle geometries proves its reliability^31–34^. Within this context, numerical modeling of underexpanded jets has been widely addressed using RANS^35^, Large Eddy Simulation LES^36,37^, and hybrid approaches, showing that even canonical axisymmetric jets feature rich dynamical structures and strong sensitivity to inlet conditions of turbulence modeling and numerical scheme selection. These developments highlight the pressing need to assess solver suitability and numerical robustness for compressible flow computations, particularly when shock resolution, computational cost, and convergence behavior become limiting factors.

An aspect of immense importance in the numerical simulation is the selection of the solver scheme, as it can significantly affect the computational cost. In computational studies, density-based DB solver has been used for modeling compressible supersonic flows, while pressure-based PB solver is specifically used for modeling incompressible subsonic flows. Because of its lower computational cost compared to the DB solver, the PB solver has been used to model number of compressible supersonic flow cases^38–41^. These studies demonstrated that PB solver can be successfully implemented to model compressible supersonic flows and to accurately predict the strength of the shock. In a different study, the performance of a gas-liquid ejector was investigated using commercial software^42^. It was found that both simulation results and experimental measurements agreed well. Different CFD models and solvers were used for the evaluation of the performance of a steam ejector^43^. The study revealed that both turbulence models SST k–ω and k–ε give comparable results. Comparative numerical studies have been performed to compare the effectiveness of pressure- and density-based solvers. For instance, Yu et al.^44^ compared the suitability of PB and DB solvers available in OpenFoam to simulate the flow inside an overexpanded single expansion supersonic ramp. The study revealed that both solvers can successfully predict shock wave structures and flow separation. However, the density-based solver showed slight differences compared with the experimental data, especially in the subsonic regions. Moreover, Singh and Mukhopadhyay^45^ investigated both PB and DB solvers to model a scramjet using ANSYS-FLUENT. The study showed that the tested solvers were in good agreement with experimental measurements. However, the PB solver outperformed the DB solver in terms of the computation cost.

Despite the comprehensive investigations of CFD tools in modeling the high-speed compressible flows, the previous studies revealed little attention has been paid comparing the accuracy of ANSYS Fluent’s built-in Pressure-Based PB solver with the supersonic flow-specialized Density-Based DB solver scheme, specifically in simulating flow downstream of underexpanded jet discharged from convergent nozzles. To address this issue, the present study provides a thorough evaluation of both schemes over a wide range of nozzle pressure ratios PRs ranging from 1.92 to 5. These pressure ratios were chosen to examine both fully and underexpanded flow conditions. The precision of the two schemes was evaluated using discharge coefficient, and qualitatively validating simulation results through Schlieren Technique. In addition, a detailed analysis of local flow properties including static pressure, Mach number, and turbulence kinetic energy was conducted to study the strength and limitations of the investigated schemes. This study provides useful information about the capabilities of PB solver compared to DB solver in modeling complex supersonic jet behavior, serving as a valuable guidance for researchers on cutting the modeling cost of compressible high- speed aerodynamics.

## Methodology

### Experimental setup

To validate the numerical model in the current study, experimental study was first performed. The Schlieren visualization technique was utilized to visualize the flow and capture shock wave structures in the near-field region of a converging nozzle. The experimental setup is divided into two sets. The first set includes a converging nozzle and a pressurized air supply tank, while the second set represents the photography setup. The nozzle used in the flow visualization tests and the numerical simulations has a fixed geometry. The inlet and the exit diameter of the nozzle is Din = 25.4 mm and De = 6 mm, respectively. In addition, the nozzle has a total length of 74 mm, which is composed of two parts, namely constant area entry section of 25 mm in length and a converging section with a length of 49 mm. The compressed air was delivered to the nozzle from a large pressurized storage tank which has a diameter of 800 mm and a height of 1640 mm. During testing, the tank was charged using an air compressor, reaching a maximum total pressure of 5 bar.

**Fig. 1:**
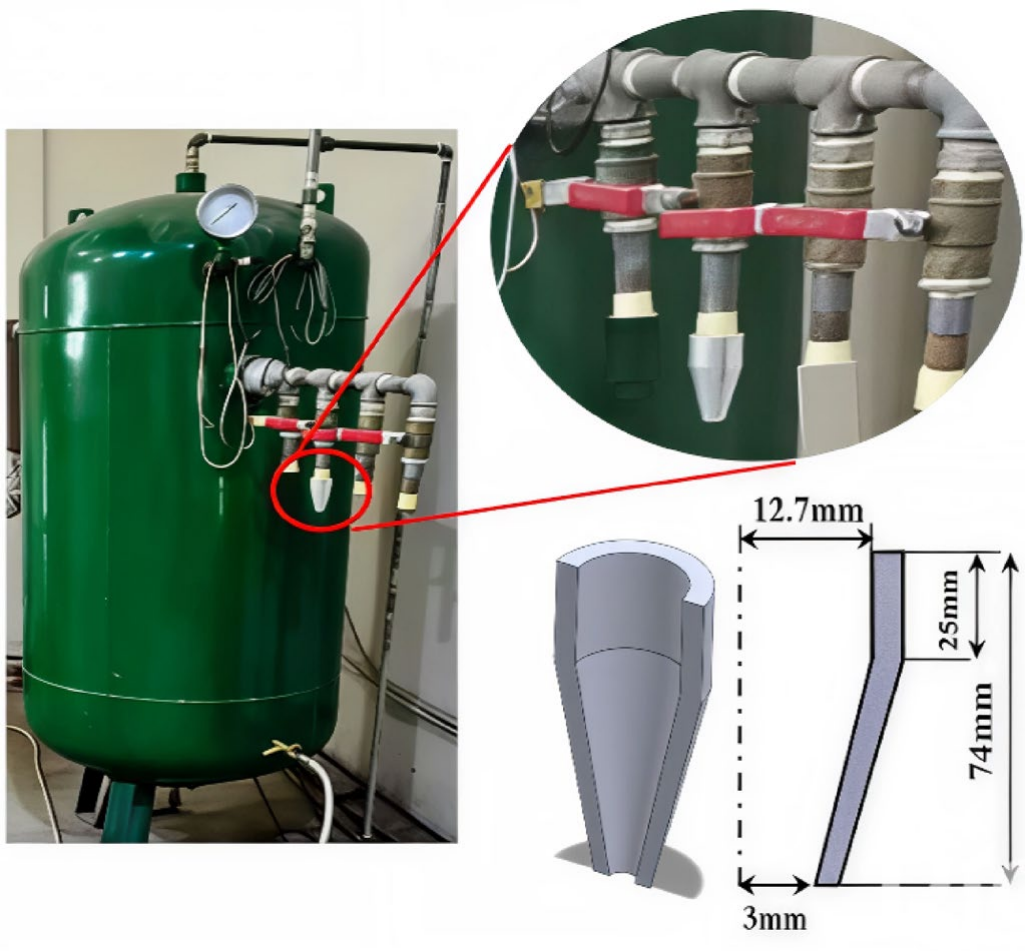
Air storage tank and nozzle dimensions.

The tank is connected to the nozzle via 25.4 mm diameter steel piping, and the flow of air discharged from the tank to the nozzle is regulated through a ball-type valve. Figure 1 illustrates the pressurized tank with the dimensions of the attached nozzle. For converged nozzles, nozzle pressure ratio is of significant importance. The state of the nozzle can be identified from the nozzle pressure ratio$PR$. Nozzle $PR$ can be calculated using the following equation:

1 $${\mathrm{PR}} = \frac{{{\mathrm{P}}_{o} }}{{{\mathrm{P}}_{a} }} = ~\left( {1 + ~\frac{{\gamma - 1}}{2}~{\mathrm{M}}^{2} } \right)^{{\frac{\gamma }{{\gamma - 1}}}}$$

where ${P}_{o}$, ${P}_{a},and\gamma$ is the total pressure, atmospheric pressure, and the ratio of specific heat for air which equal to 1.4, respectively. If the nozzle is choked, the nozzle will be fully expanded and Mach number at the exit M = 1. The pressure of the jet at the exit in this case is known as the critical Pressure Ratio${PR}_{c}$. Therefore Eq. 1 can be rewritten for a choked nozzle with exit Mach number M=1as follows:

2 $${\mathrm{PR}}_{c} = \frac{{{\mathrm{P}}_{o} }}{{{\mathrm{P}}_{c} }} = ~\left( {~\frac{{\gamma + 1}}{2}~} \right)^{{\frac{\gamma }{{\gamma - 1}}}}$$

where ${P}_{c}$is critical pressure. Therefore, in the current study, the critical value of the pressure ratio${PR}_{c}$ when the nozzle is choked with exit Mach number M = 1 and ${\gamma}_{air}=1.4$ equal to ${PR}_{c}=1.89.$.

In the current investigation, four values of nozzle PRs were tested; these are PR = 1.92, 3,4, and 5. These ratios were chosen such that two nozzle conditions can be investigated. The first one is when nozzle PR is close to the critical ratio${PR}_{c}=1.89.$ In this case, the exit air pressure close to the atmospheric back pressure. As a result, the nozzle is choked and the jet stream is fully expanded outside the nozzle with exit Mach number M = 1. On the other hand, when nozzle PR is higher than the critical value PR = 3, 4, and 5, the exit pressure becomes greater than the atmospheric pressure, thus the jet underexpands downstream the nozzle exit plane and series of diamond shocks occurs in the jet plume.

**Fig. 2:**
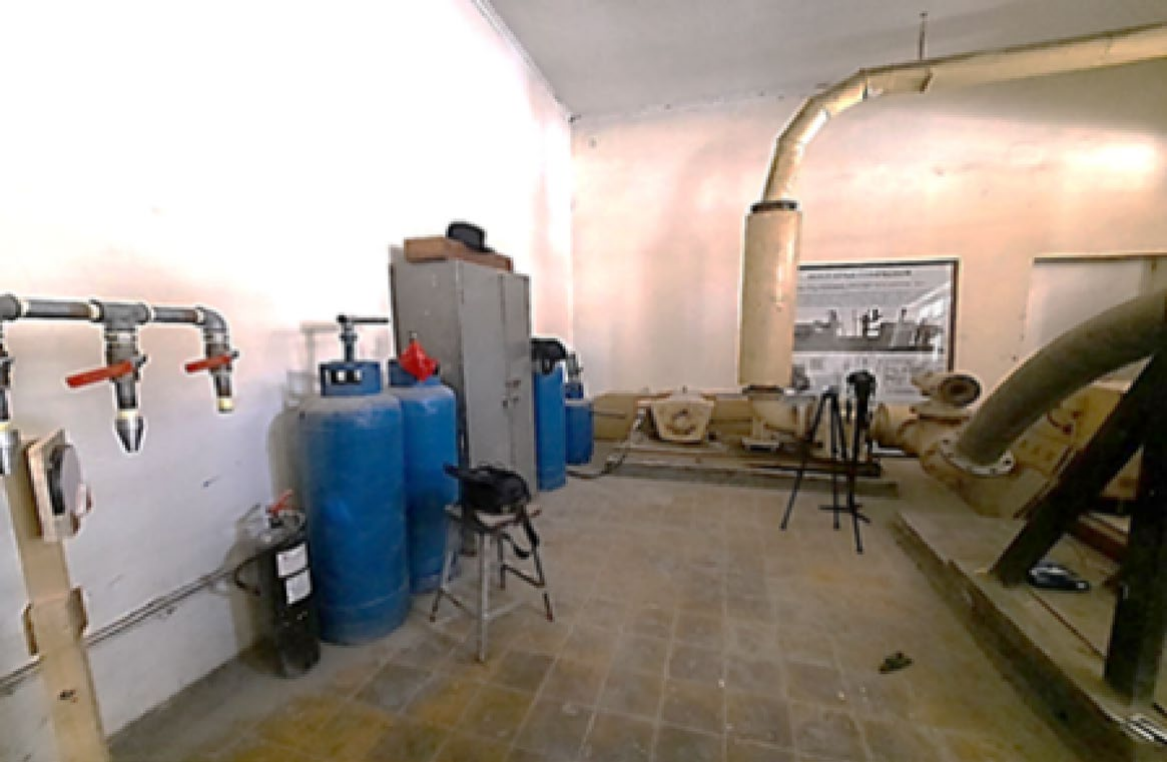
Schlieren optics arrangement.

The second part of the experimental setup is the Schlieren optics that was used for capturing the flow structures outside the nozzle. The Schlieren visualization setup is composed of concave mirror, Canon camera, knife edge, and point source LED light. A standard concave mirror with an approximate diameter of 148 mm was utilized. The mirror possesses a focal length of approximately 1500 mm, resulting in a radius of curvature (2f) of about 3000 mm. The mirror was mounted on a home-made wooden stand designed to enable precise alignment with the optical axis. In addition, to ensure a stable and coherent light source for the optical testing, bright point light source is constructed using a simple home-built LED circuit. The LED is soldered onto Vero board which has a size of 3 cm × 7 cm. To regulate the current supplied to the LED light, a 220 Ω resistor was used. Moreover, this light source was powered by a 9 V battery. A Canon digital camera was used to capture the reflected image. The camera is positioned along the optical axis at the appropriate distance behind the knife edge which was used to create the required contrast which reveals the shock waves. A standard razor blade was utilized which was fixed on a tripod. This setup allows height adjustment to ensure that the edge can be precisely aligned. Figure 2 illustrates Schlieren optics setup.

### Numerical model

In the numerical simulation, the flow downstream a converging nozzle was numerically investigated using ANSYS-Fluent commercial software. Two different solver schemes were utilized in the computation process to compare their accuracy on the flow structures downstream the converging nozzle, namely Pressure-Based (PB) and Density-Based (DB) solver schemes. The converging nozzle dimensions are similar to the nozzle used in the flow visualization tests. Based on the nozzle PR, the nozzle is carefully designed such that the jet outside the nozzle is underexpanded when PR > 1.92 and it is fully expanded when PR ≤ 1.92.

**Fig. 3:**
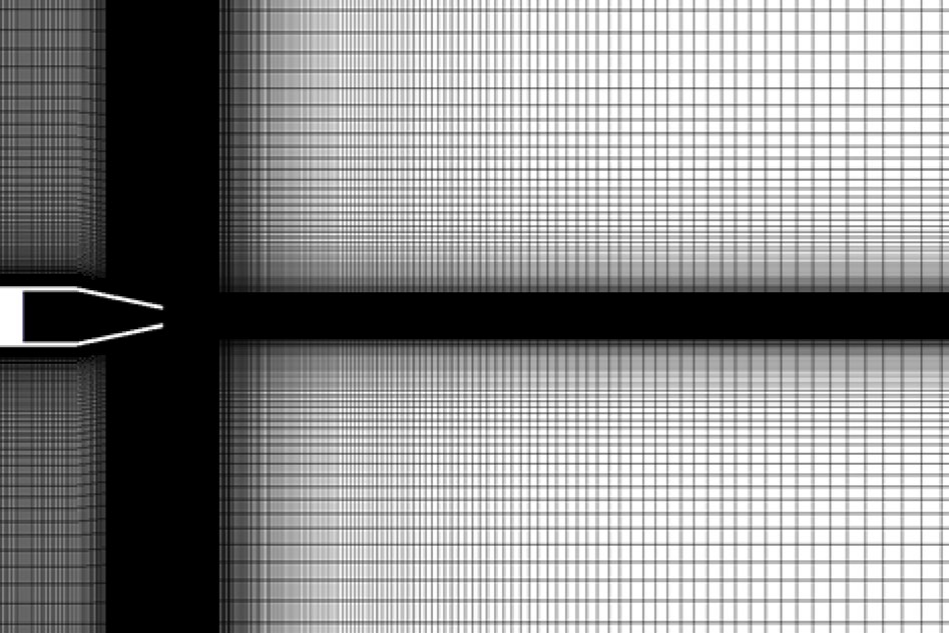
Meshed domain.

The flow domain regions inside and outside the nozzle were discretized using Ansys meshing tool. A rectangular domain divided into 11 subdomains was designed to generate a structured grid around and inside the nozzle. The height of the domain above the nozzle is 27De, while the domain length downstream the nozzle exit plane extends 75De to ensure stable solution and to avoid backflow. Due to the simplicity of the nozzle geometry in the current work, uniform mesh with quadrilateral elements was considered inside and outside the nozzle. In order to capture the shock structures in the underexpanded jet plum, the region that extends from the exit of the nozzle to the end of the domain was carefully treated by increasing the number of elements especially in the shear layer regions and the expected locations where the shock waves could occur. Figure 3 shows the meshed domain inside and outside the nozzle. To ensure that the used mesh has a good quality, a mesh independence study was conducted at nozzle PR = 4. The effect of four different mesh sizes on the mass flow rate through the nozzle was examined as illustrated in Table 1. It can be seen that increasing the number of elements beyond 228,320 has a minor effect on the mass flow rate passing through the nozzle. Therefore, the total number of elements that was considered in the current work was 228,320.

**Table 1:** Mesh Independence.

Number of elements	Mass Flow rate, [kg/s]
40,000	0.0119
146,800	0.0123
228,320	0.01241
279,200	0.01244

In the numerical analysis, the steady Reynolds Average Navier-Stokes RANS equations were solved using ANSYS-FLUENT. The flow is considered 2D, steady, compressible, axisymmetric and viscous. The compressible steady state RANS equations represented in tensor format that were used in the current study can be described as follows:

1. Continuity
  3 $$\frac{{~\partial }}{{\partial x_{i} }}\left( {\rho ~u_{i} } \right) = 0$$
2. Momentum
  4 $$\frac{{~\partial \left( {\rho ~u_{i} u_{j} } \right)}}{{\partial x_{i} }} = - \frac{{\partial P}}{{\partial x_{i} }} + ~\frac{{~\partial \tau _{{ij}} }}{{\partial x_{j} }}$$
3. Energy
  5 $$\frac{{~\partial }}{{\partial x_{i} }}\left( {u_{i} \left( {\rho ~E + P} \right)} \right) = \nabla \left( {\lambda \frac{{\partial T}}{{\partial x_{i} }} + ~u_{i} ~\tau _{{ij}} } \right)$$

**Fig. 4:**
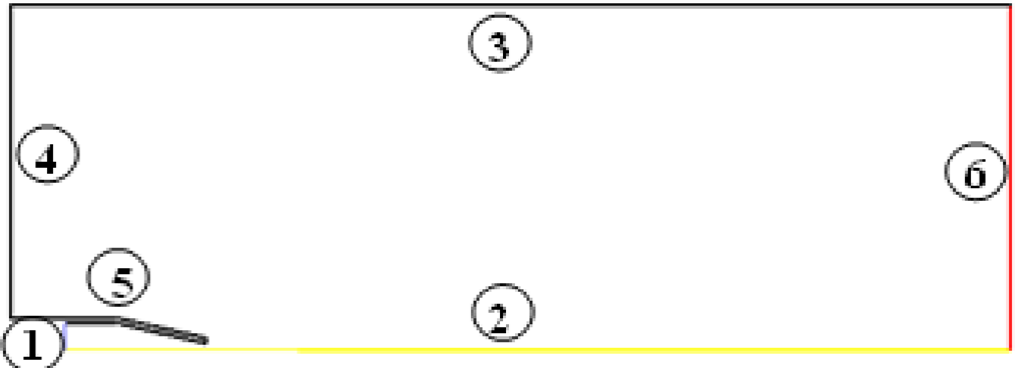
Boundary conditions: (1) nozzle inlet, (2) symmetry, (3 and 4) farfield, (5) wall, and (6) pressure outlet.

In the current case, the flow is inside and outside the nozzle is considered as isentropic. Thus, the system is insulated with no heat transfer between the system and the surrounding. In the present investigation, air is chosen as the working medium and was considered an ideal gas with a specific heat ratio γ = 1.4 and a gas constant *R* = 287 J/kg.K. Both DB and PB solver schemes were investigated and compared. When the DB solver was used, the implicit solution method was chosen, and second-order upwind scheme was selected for the special discretization of both flow and turbulent kinetic energy. On the other hand, when the PB solver was chosen, the coupled scheme was used for pressure velocity coupling, and the second-order upwind was chosen for the pressure, density, momentum, turbulent kinetic energy, and energy spatial discretization. For PB and DB solvers, the two-equation k-ε turbulence model was chosen for the simulations because it has been successfully used for modeling high speed and swirling flows, as reported in 46. The boundary conditions are illustrated in Fig. 4.

**Fig. 5:**
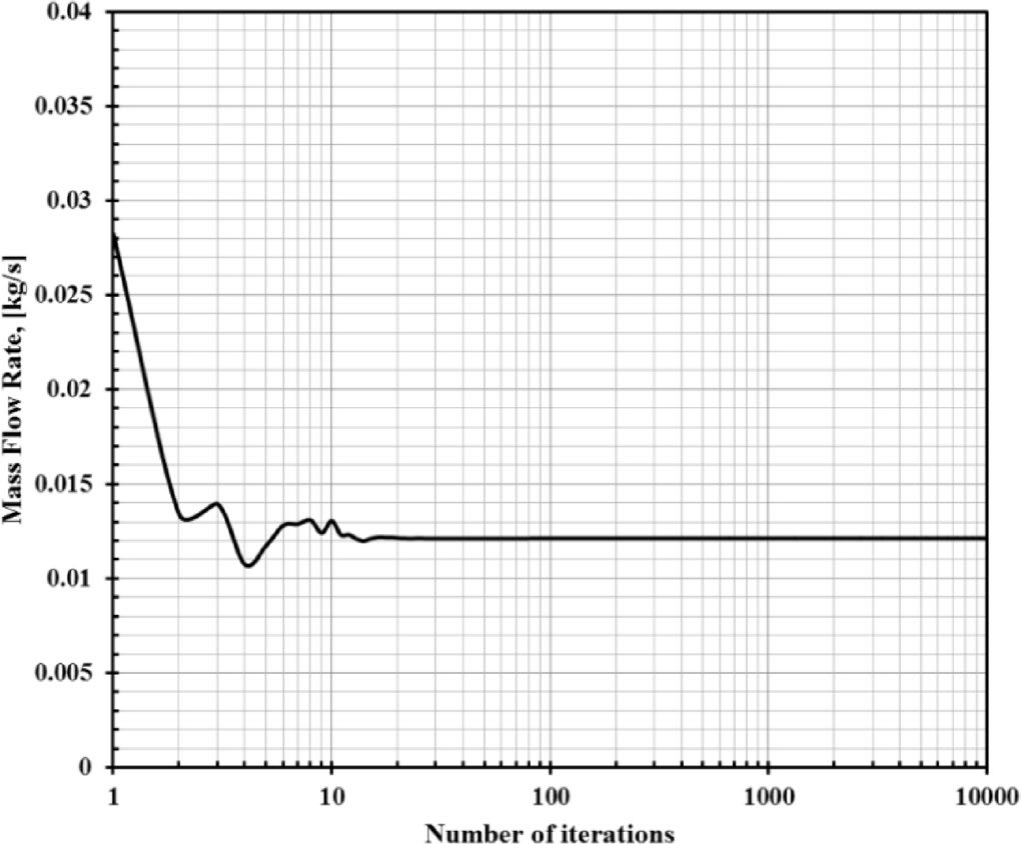
Convergence history of mas flow rate.

The nozzle inlet was set as a pressure inlet, and the initial gauge total pressure varied between 192 × 10^3^ and 500 × 10^3^ Pa. The bottom side of the domain was set as symmetry to reduce the computation time. The upper and the left sides of the domain were set as a pressure farfield. Finally, the right side of the domain was set as pressure outlet. The pressure at the outlet of the nozzle is equal to 101,325 Pa. Table 2 summarizes the details of the adapted boundary conditions in the current investigation, while Table 3 presents the chosen solution and control method for the investigated case. Moreover, in order to check the convergence of the numerical simulation, the history of mass flow rate vs. the number of iterations is illustrated in Fig. 5. From Fig. 5, it can be seen that the mass flow rate value was converged after 10,000 iterations. Therefore, all the simulations were considered converged after 10,000 iterations with a residual error value equal to 10 − 8.

**Table 2:** Boundary conditions.

Index	Domain name	Boundary condition	Value x 10^3^
1	Nozzle inlet	Pressure inlet [Pa]	500, 400, 300,192
2	Symmetry line	Symmetry	-
3 and 4	Top and left vertical line	Farfield	-
5	Nozzle surface	Wall	No-slip
6	Outlet	Pressure [Pa]	101,325

**Table 3:** Solution method and control.

Description	Density-based solver	Pressure-based solver
Solution Method	Implicit	Coupled/pressure and velocity
Special discretization	2nd Order Upwind	2nd Order Upwind
Solution control	Courant Number = 200	Courant Number = 200
Turbulence model	Standard k-ɛ	Standard k-ɛ
Turbulent kinetic energy	0.8	0.8
Turbulent dissipation	0.8	0.8
Turbulent viscosity	1	1

###  Solver convergence and computational cost analysis

**Fig. 6:**
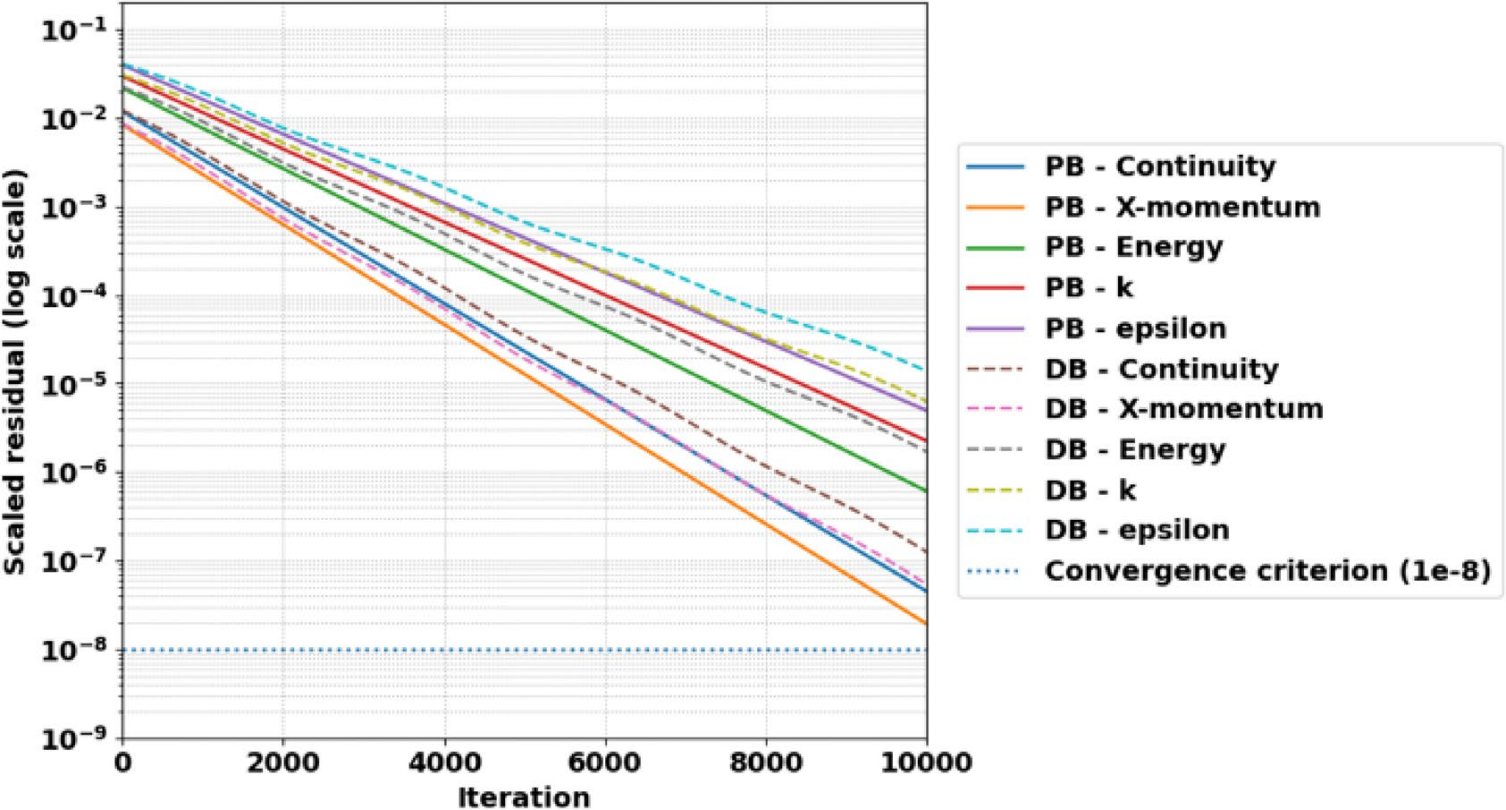
Residual convergence history comparison between PB and DB solvers at PR = 4.

To quantitatively evaluate the computational efficiency of the investigated solvers, convergence histories and CPU times were recorded for all simulations. Figure 6 illustrates the residual decay for continuity, momentum, and energy equations at PR = 4. Both solvers achieved the prescribed convergence criterion of 10⁻⁸; however, the pressure-based solver exhibited faster residual decay and smoother convergence behavior. Table XX presents a quantitative comparison of convergence performance and computational cost between the investigated solver schemes at PR = 4. Although both solvers required 10,000 iterations to satisfy the prescribed residual threshold of 10⁻⁸, the pressure-based solver demonstrated lower computational expense. The total CPU time for the PB solver was 118 min compared to 151 min for the DB solver, corresponding to a computational time reduction of approximately 22%. This confirms the improved numerical efficiency of the PB scheme while maintaining comparable prediction accuracy for shock structure and local flow parameters.

**Table 4:** Convergence performance and computational cost comparison between pressure-based (PB) and density-based (DB) solvers at PR = 4 (identical mesh and hardware conditions).

Solver	Total iterations	Residual threshold	CPU time (min)	Average time per 1000 iterations (min)
Pressure-Based (PB)	10,000	1 × 10⁻⁸	**118**	11.8
Density-Based (DB)	10,000	1 × 10⁻⁸	**151**	15.1

#### Discharge coefficient (${\mathbf{C}}_{\mathbf{D}})$

To evaluate the performance of the convergent nozzle, an important parameter, discharge coefficient${\mathrm{C}}_{\mathrm{D}}$, was numerically calculated. The discharge coefficient ${\mathrm{C}}_{\mathrm{D}}$ can be defined as the ratio between the mass flow rate to the maximum mass flow rate when the nozzle is choked and flow is isentropic^12^, the ${\mathrm{C}}_{\mathrm{D}}$. can be calculated as follows:

6 $$C_{D} = ~\frac{{\dot{m}}}{{\dot{m}_{{ideal}} }}~$$

where $\dot{\mathrm{m}}$ and are the mass flow rate and the maximum mass flow rate. The maximum mass flow rate can be calculated as follows^12^:

7 $$\dot{m}_{{ideal}} = ~\frac{{\rho _{o} A_{c} }}{{\left( {RT_{o} } \right)^{{\frac{1}{2}}} }}~\left[ {\frac{{2\gamma }}{{\gamma + 1}}~\left( {\frac{2}{{\gamma + 1}}} \right)^{{\frac{2}{{\gamma - 1}}}} } \right]^{{\frac{1}{2}}}$$

### Validation of numerical model

**Fig. 7:**
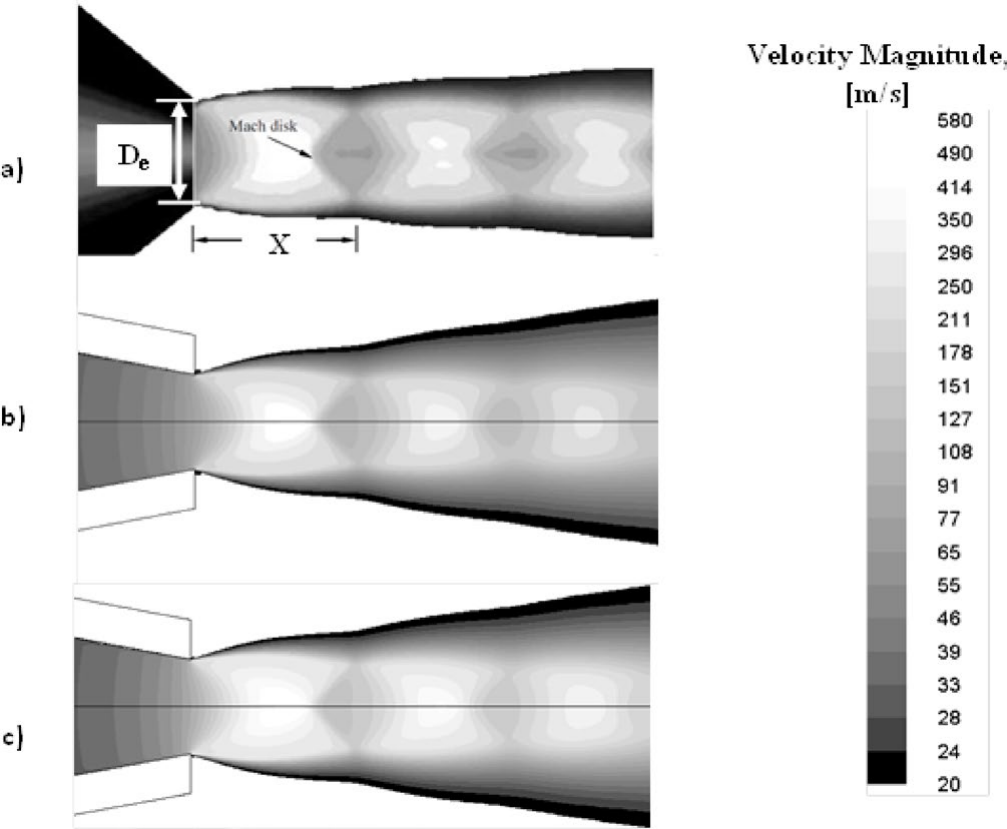
Contours of velocity magnitude: (**a**) experimental study^11^, (**b**) current study PB solver, and (**c**) current study DB solver, at nozzle PR = 4.

The numerical model in the current investigation was validated with an experimental study reported by^11^. In this study, the impingement effect of an underexpanded jet stream discharged from a converging nozzle on a flat plate was investigated. The reported results at nozzle PR = 4 were compared with the current numerical study using PB and DB solvers. Figure 7 shows a comparison of velocity magnitude contours between the current numerical study and the experimental work conducted by^11^. From Fig. 7, the result of the current numerical study using PB and DB solvers and the reported experimental measurements have consistently demonstrated that the nozzle at this pressure ratio is underexpanded, which can be identified by clear shock wave reflections downstream the nozzle. Furthermore, in^11^, the distance from the nozzle exit to the first shock cell (Mach disk) was X/De = 1.6, as shown in Fig. 7-a. However, in the current numerical investigations the Mach disk occurs at a distance of 1.57 De for both PB and DB simulations as illustrated in Figs. 7-b and c, respectively. Despite the differences in the approaches used in the PB and DB solvers, the results were consistent, with only a slight difference compared to the reported experimental data in^11^.

**Fig. 8:**
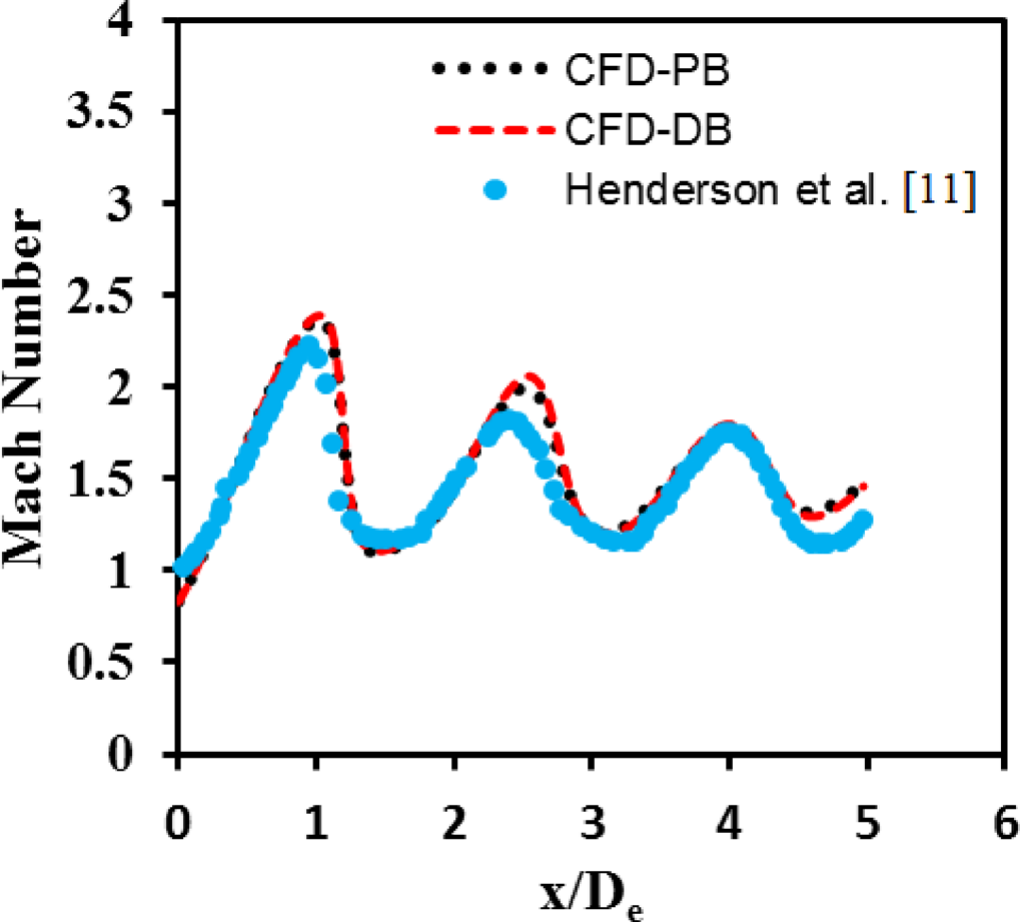
Validation of axial Mach number with data reported in^32^, at PR = 4.

Moreover, the axial Mach number distribution along the centerline of the jet plume at nozzle PR = 4 obtained from the current numerical simulation was validated with the reported data in [11], as illustrated in Fig. 8. The data was plotted from the exit plane of the jet X/De = 0 to X/De = 5. From Fig. 8, an obvious fluctuation in the Mach number distribution can be seen. The flow outside the nozzle accelerates until it reaches M = 2.5, and then it decelerates to a lower supersonic Mach number. This behavior of the flow is due to the presence of expansion waves near the nozzle exit plane. These waves occur because of the difference between jet exit pressure and the ambient pressure (jet pressure is higher than the atmospheric pressure). In addition, it can be seen that the strength of the shocks reduces as the flow advances further downstream the nozzle exit lip. Although a perfect agreement between the predicted Mach number using PB and DB solvers can be seen, the current simulation over-predict the maximum value of the Mach number compared to the experimental measurements with a maximum error of 4.5%. This deviation in Mach number values can be attributed due to the difference in the nozzle size, which is smaller than the one used in the experimental measurements reported in [32].

## Results and discussion

### Schlieren visualization vs simulation

Using experimental Schlieren visualization and numerical simulation, the capabilities of the two investigated PB and DB solver schemes in capturing the evolution of the flow field downstream of the converging nozzle were compared. The experimental flow visualization was conducted using the same nozzle design that was used in the simulations. The Schlieren technique, which visualizes gradients in the density, provides a qualitative assessment of compressible flow features, while the numerical results offer a more quantitative visualization of the density field. The bright regions in the numerical plots correspond to zones of high density, while the darker regions indicate low-density flow areas.

**Fig. 9:**
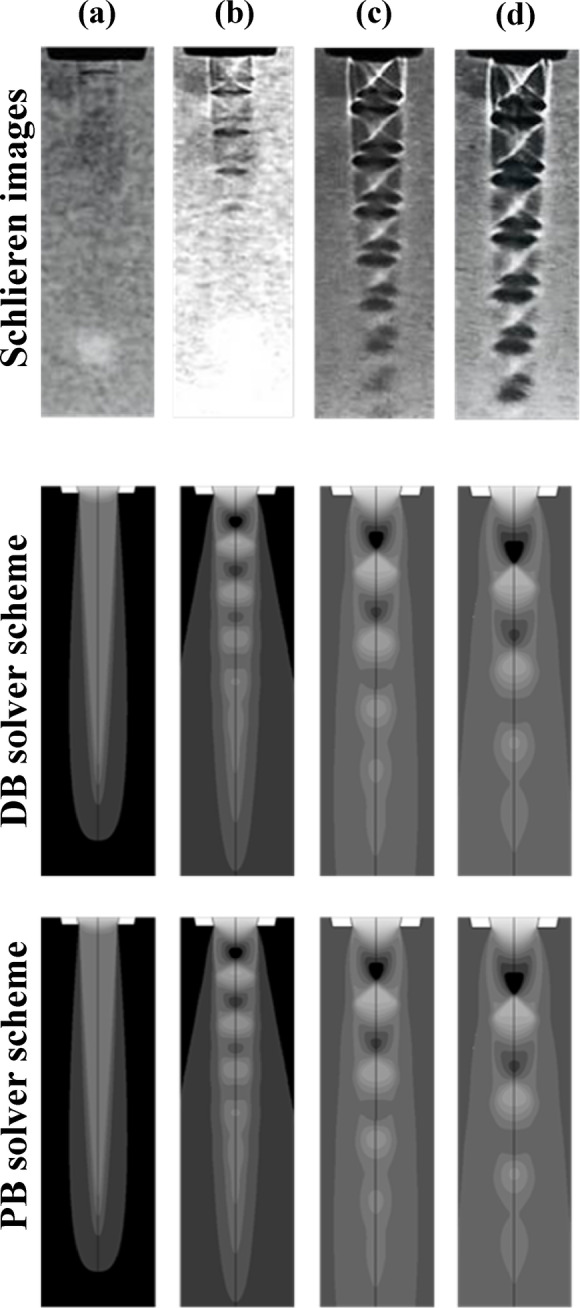
Schlieren imaging (Upper row) compared with simulation (Lower row) at PR: (**a**) 1.92, (**b**) 3, (**c**) 4, and (**d**) 5.

Figure 9 illustrates a comparison between the experimental Schlieren images of flow downstream the nozzle and the density contour plots obtained from numerical simulation using DB and PB solver Schemes for pressure ratios PR = 1.92 to 5. These pressure ratios were selected to represent a progressive increase in the level of underexpansion, allowing for detailed observation of shock formation and development. At the lowest tested PR = 1.92, a fully expanded region downstream the converged nozzle can be seen in the Schlieren and numerical simulation. In addition, a region of constant density higher than the ambient one can be observed in the core region of the nozzle, as shown in Fig. 9-a.

As the nozzle PR increases to 3, the nozzle becomes moderately underexpanded with diamond shock waves structures can be seen after the nozzle exit edge. The density contours obtained from numerical simulation confirm the formation of compact shock structures behind the nozzle exit lip, with first cell occurs at X/De = 1.09. A repeated pattern of dark, low-density region before the shock and bright, high-density region after the shock can be seen, as illustrated in Fig. 9-b. This confirms the formation of expansion waves which naturally reduce the flow pressure and density after the shock and increase flow velocity. It can be seen that both DB and PB solvers can correctly resolve the near field region and capture the train of alternating shock cells downstream the nozzle. At PR = 4, more pronounced shock structures appear in the jet. The Schlieren image shows a well-organized sequence of oblique shocks and expansion waves, forming a periodic shock cell structure downstream the nozzle. The PB and DB solvers successfully capture these features in the numerical density contours, with increased gradient intensity and clearer definition of the shock-expansion wave cells compared with PR = 1.92. In addition, an obvious increase the cell size of the shock wave can be observed with first cell occurs at about X/De = 1.57, as shown in Fig. 9-c. In general, at this pressure ratio, both solvers predict well the expansion and compression shock wave series downstream the nozzle when compared with experimental flow visualizations. At nozzle PR = 5 it can be seen that density variation becomes more pronounced and the shock cell pattern becomes more intense and complex. In addition, the cell size becomes bigger with higher density gradient. Moreover, the first shock cell occurs at about twice the jet diameter, as can be seen in Fig. 9-d. In general, as the nozzle PR increases, both the strength and the size of the first shock cell increases; this agrees well with previous work reported in^6–9^. Both DB and PB solver still captures the overall structure and dynamics of the underexpanded jet accurately. Despite the complex phenomena that is associated with flow downstream underexpansed nozzle, it can be noticed that PB solver scheme predicts well the region downstream the nozzle compared with DB solver.

### Flow structure downstream the nozzle

**Fig. 10:**
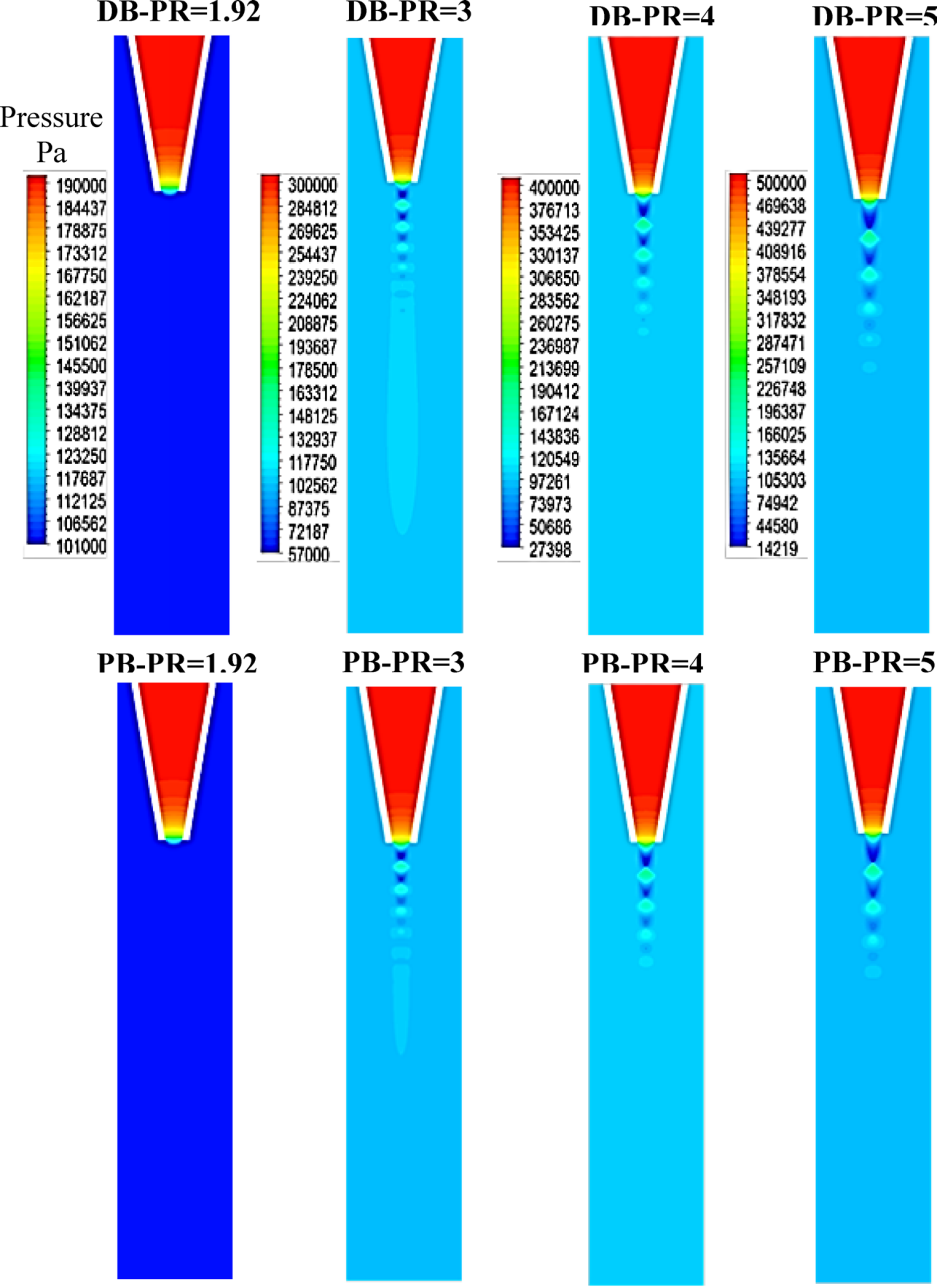
Contours of pressure for DB and PB solvers at nozzle PR = 1.92, 2, 3, 4, and5.

**Fig. 11:**
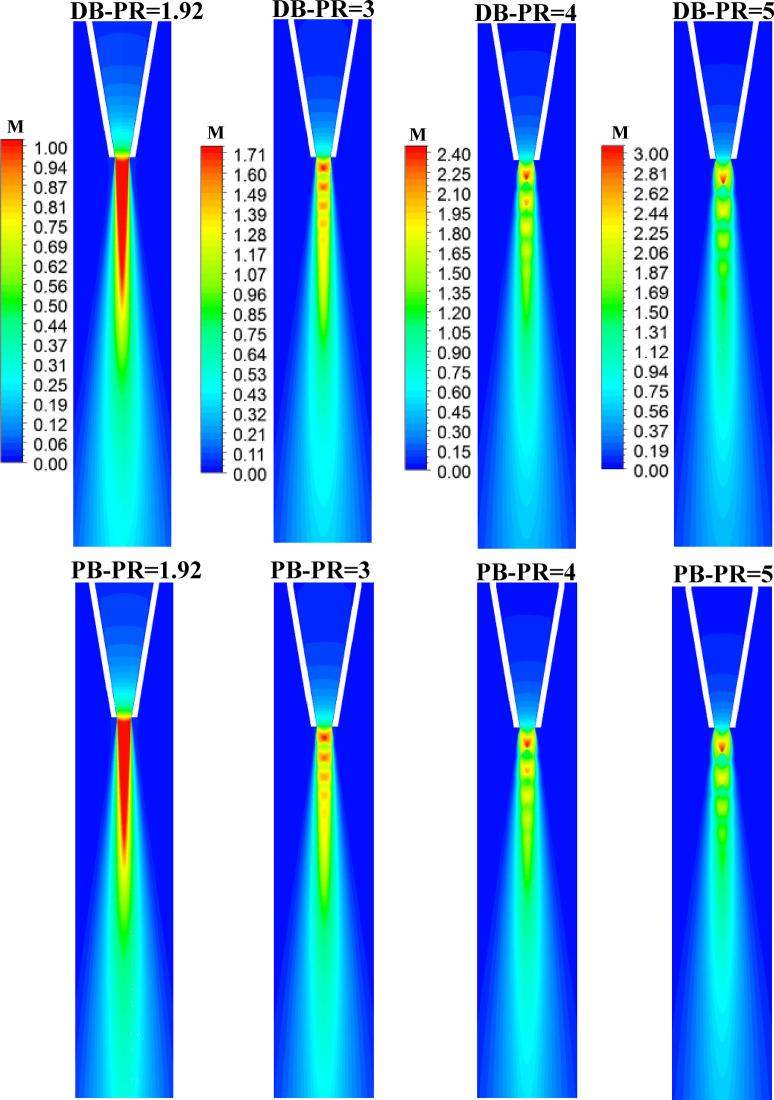
Contours of Mach number for DB and PB solvers at nozzle PR = 1.92, 2, 3, 4, and 5.

In this section, the pressure, velocity, and turbulent kinetic energy contours obtained from numerical simulation using DB and PB solvers at four different nozzle pressure ratios ranging from 1.92 to 5 are compared. Figure 10 illustrates pressure contours for PB and DB solvers at different nozzle pressure ratios. It can be seen that at nozzle PR = 1.92, both solvers clearly show that the nozzle pressure at the exit does not exceed the ambient pressure, thus the flow does not expand outside the nozzle and no shock diamonds can be seen. As the nozzle PR increases to 3, the jet pressure at the exit of the nozzle becomes larger than the atmospheric pressure. Therefore, the nozzle becomes underexpanded, and a series of low- and high-pressure reigns can be seen. In addition, both solvers reflect the same flow behavior downstream the nozzle. When increasing nozzle PR to 4 and 5, the effect of pressure rise becomes stronger and the flow expands more downstream the exit plane of the nozzle, and the distance between successive high and low regions becomes larger, which is mainly due to the increase in the pressure difference between the jet exit pressure and the atmospheric pressure. The greater the pressure difference, the higher the strength of the shock cells downstream the nozzle exit plane. Figure 11 illustrates the contours of Mach number downstream the nozzle at the investigated nozzle pressure ratios. At PR = 1.92, the flow expended downstream the nozzle lip and the flow Mach number reaches M = 1 at the exit of the nozzle which indicates that the nozzle is chocked. In addition, both the PB and DB solvers successfully resolve the region downstream the nozzle with no significant difference. At nozzle PR = 3, the flow accelerates outside the nozzle, creating a supersonic region with a Mach number of M = 1.7 ahead of the first shock cell, followed by a lower supersonic region. This diamond shock structure is consistent with the behavior observed in Fig. 10. Furthermore, the first shock cell, Mach disc, occurs at a sgtreamwise location X/De = 1.09 downstream the nozzle lip. At nozzle PR = 4 and 5, an obvious increase in the strength and the location of the first shock cell occurs downstream the nozzle exit. The higher the pressure ratio, the stronger the shock is, and the further the distance of the first shock cell occurs. For instance, at PR = 4, the flow upstream the Mach number reaches M = 2.4 with the location of the first shock shifted further downstream to X/De = 1.59. At the highest investigated pressure ratio PR = 5, the shock strength further increases as the upstream Mach number increases to M = 3. The first shock cell occurs at a distance equal to about X/De = 2. Finally, there was a perfect agreement between the results obtained from the PB and DB solvers. To further illustrate the differences between the solvers, the local distribution of flow quantities will be presented in Sect.  3.3.

### Local flow parameters

**Fig. 12:**
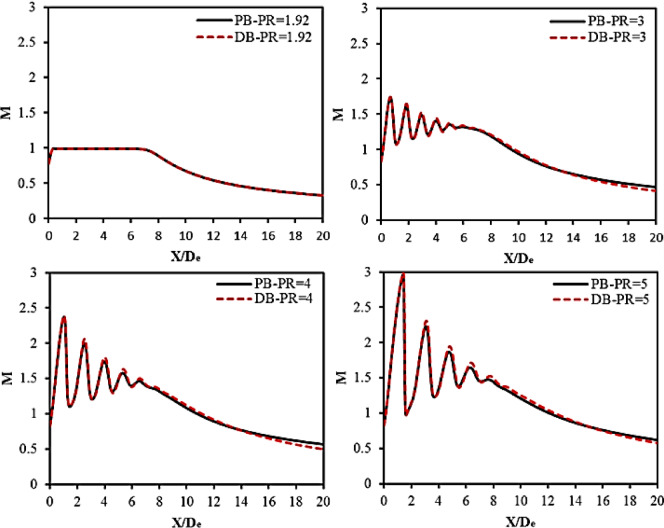
Mach number distribution along the jet centerline at nozzle PR = 1.92, 3, 4, and 5.

This section illustrates the distribution of local flow quantities along the centerline of the jet plume, including Mach numbers and turbulent kinetic energy. These flow parameters were drawn at a distance of 20De from the nozzle exit edge. The distribution of Mach number along the jet centerline was examined at PR = 1.92, 3, 4, and 5, as demonstrated in Fig. 12. From Fig. 12, one can notice that, at the lowest tested nozzle pressure ratio PR = 1.92, the flow reaches the speed of sound. This indicates that the nozzle is fully expanded, leading to the formation of a potential core with a Mach number of M = 1 behind the nozzle exit edge, because the flow exits the nozzle with a pressure equal to the ambient pressure. At X/De = 7, the flow starts decelerating to a subsonic flow, indicating the end of the potential core. On the other hand, at higher nozzle pressure ratios, PR = 3, 4, and 5, severe fluctuation in the flow can be seen near the exit, and then it decays at X/De = 8 downstream the nozzle exit edge. This behavior can be attributed to the acceleration of the flow downstream the nozzle. The flow accelerates to a supersonic speed, and then it gently becomes subsonic after a streamwise distance of 8De. This oscillation in flow Mach number clearly confirms the presence of the diamond shock wave cell structures downstream the nozzle that was previously illustrated in Fig. 11. The maximum Mach numbers that the flow reaches upstream the first shock wave at PR = 3, 4, and 5 are 1.7, 2.4, and 3, respectively. Therefore, it can be concluded that as the nozzle pressure ratio increases, the flow Mach number ahead of the shock wave increases as well, leading to a stronger shock wave. In addition, the results obtained using PB and DB solvers are perfectly agreed. A slight difference can be seen at X/De = 2, where the DB solver underestimates the flow speed. This behavior is expected in this region, as the DB solver performs better at supersonic regions than subsonic regions.

**Fig. 13:**
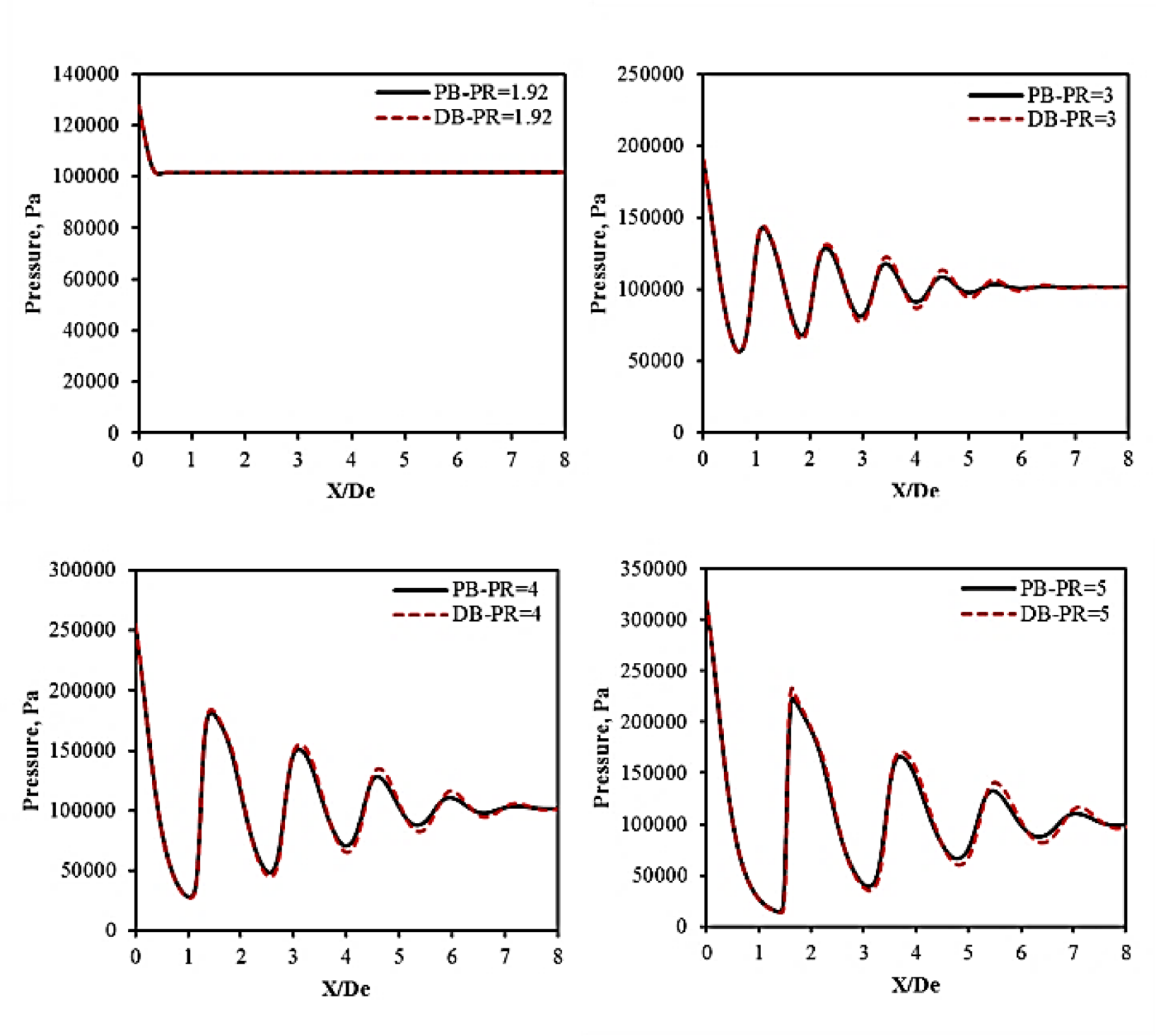
Pressure distribution at nozzle PR = 1.92, 3, 4, and 5.

The streamwise pressure variation along the centerline of the jet downstream of the converging nozzle using PB and DB solvers is depicted in Fig. 13. At nozzle PR = 1.92, the pressure drops sharply near the nozzle exit which indicates that the flow accelerates downstream the nozzle until it reaches a speed equal to the local speed of sound as previously seen in Fig. 11. In addition, both PB and DB schemes show close agreement.

At nozzle PR = 3, oscillatory pressure profile can be seen. These oscillatory pressure profile decays at about a streamwise distance 5 times the jet exit diameter. This indicates that the jet is underexpanded because of the pressure difference between the nozzle exit and the surrounding pressure, with the exit pressure exceeding atmospheric pressure, where oblique shocks and expansion fans interact downstream, causing pressure oscillations until it decays at X/De = 5.

At higher nozzle pressure ratios PR = 4 and 5, the oscillation behavior continues downstream the nozzle. However, the amplitude of the oscillation sharply increases. The higher the pressure ratio the stronger the oscillation is. This suggests that more strong shock expansion occurs downstream the nozzle. In addition, both numerical solvers can predict the amplitude of the oscillations, with slight numerical discrepancies can be seen.

**Fig. 14:**
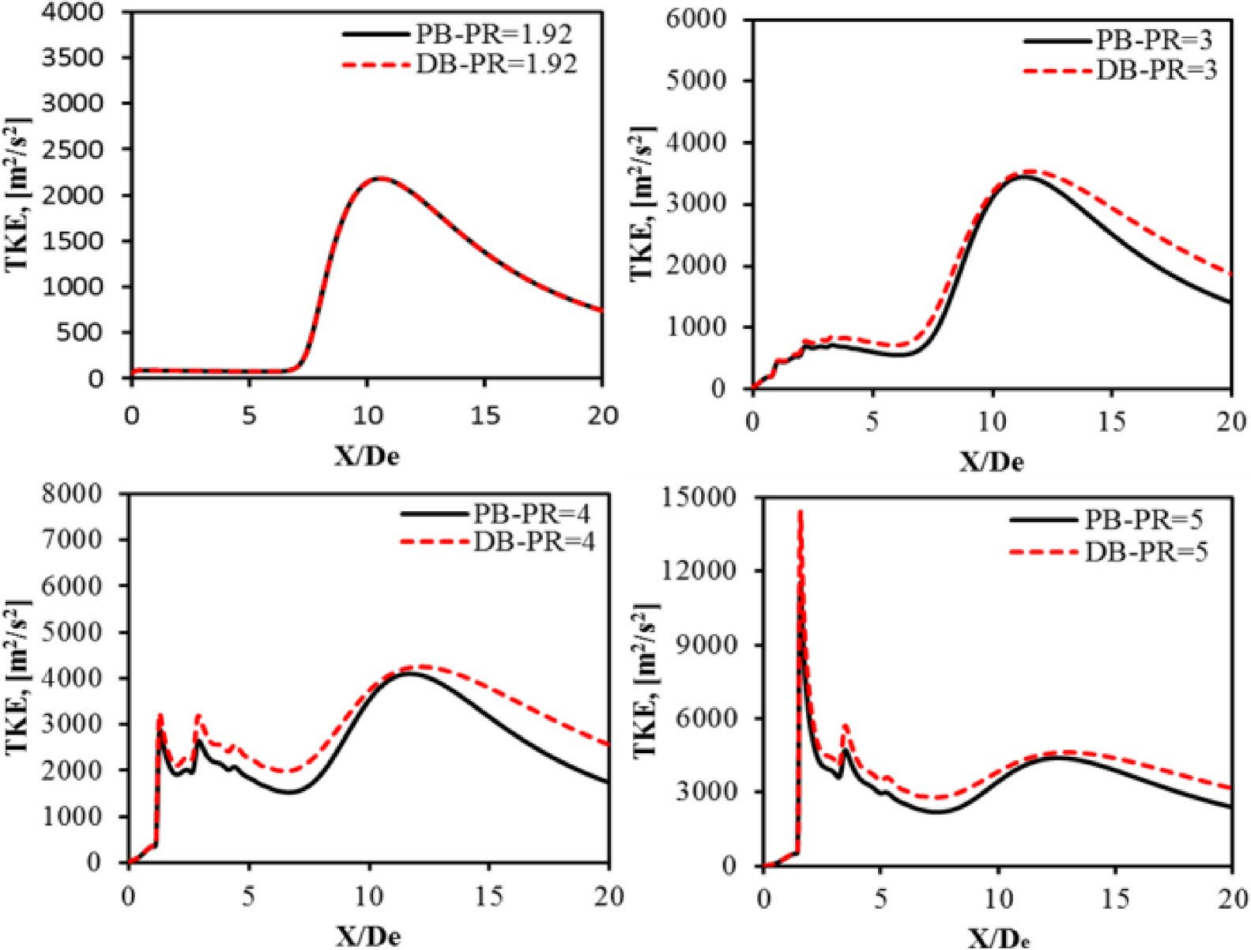
Turbulent kinetic energy (TKE) distribution at nozzle PR = 1.92, 3, 4, and 5.

Figure 14 shows the axial distribution of the turbulent kinetic energy (TKE) along the jet centerline using the PB and DB solvers at PR = 1.92, 3, 4, and 5. One can notice that the investigated PB and DB solvers show good agreement at the small pressure ratio. However, as the nozzle pressure ratio increases to PR = 3, 4, and 5, the DB solver overpredicts the turbulent kinetic energy, especially at X/De > 12. Because the flow decelerates to subsonic speed and shock wave cells decay after X/De > 12. Since the DB solver is known for its poor performance at subsonic speeds, the turbulent kinetic energy is overpredicted. In the near-field region X/De < 5, very low turbulence levels can be seen at PR = 1.92. This is because the nozzle is fully expanded at this PR and the absence of the shock wave cell structures in the near-field region. Moreover, a significant increase in the turbulent kinetic energy level can be seen at the PR = 5. This could be because of the formation of a region of strong expansion waves near the nozzle exit plane and the presence of a shear layer, which exhibits a high turbulence level due to the severe velocity gradient at this pressure ratio.

### Nozzle performance evaluation

**Fig. 15:**
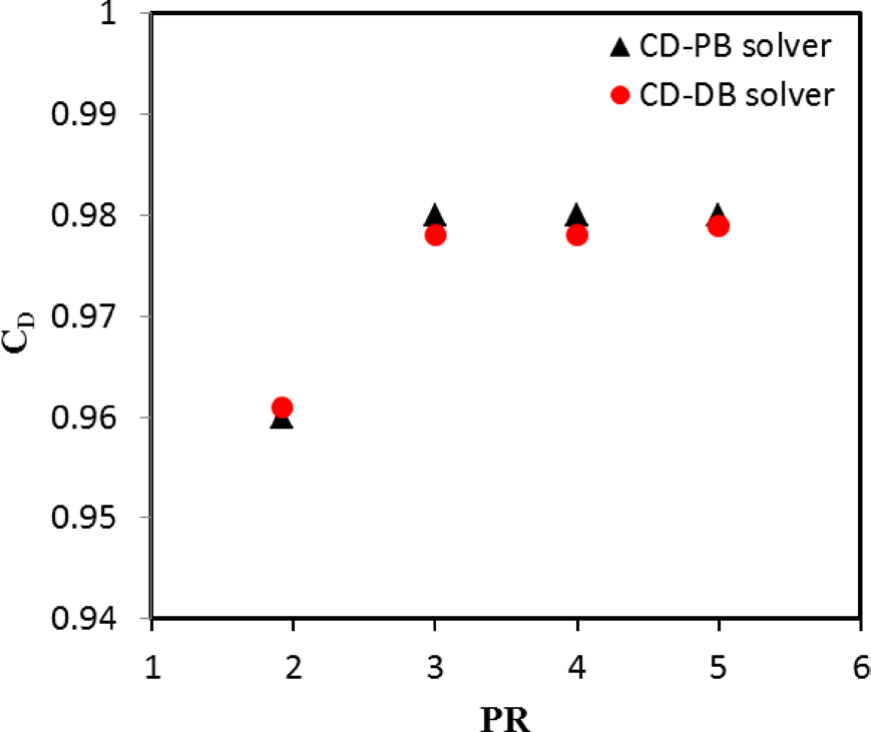
Comparison between PB and DB solvers using discharge coefficient.

The performance of the nozzle was evaluated through calculating the discharge coefficient at various nozzle pressure ratio PR = 1.92, 3, 4, and 5 as can be seen in Fig. 15. From Fig. 15, one can notice that the behavior of the nozzle discharge coefficient differs as the nozzle pressure ratio varies. For instance, at the lowest pressure ratio (PR = 1.92), the discharge coefficient predicted by both solvers is the lowest compared with the higher-pressure ratios. This indicates that flow has energy losses due to the viscous effect and boundary layer growth. At higher nozzle pressure ratio (PR > 3), the discharge coefficient constantly increases reaching to a maximum value of 0.98. This indicates that the nozzle is fully chocked and under expanded. In addition, the results revealed that a closely match between the discharge coefficients predicted by PB and DB solver schemes. The PB solver slightly over predicts the discharge coefficients with maximum error of 0.2%.

##  Conclusions

The present work investigated the suitability of two different CFD solvers, namely Pressure-Based PB and Density-Based DB solvers to predict the flow structures downstream a convergent nozzle at different nozzle PR. The PR was varied between 1.92 and 5, which correspond to fully expanded nozzle and moderately underexpanded nozzle, respectively. The current numerical results were validated with available literature and experimental Schlieren flow visualization. The numerical study and flow visualization results clearly showed that at PR = 1.92, the nozzle is choked, perfectly expanded with a potential core flow Mach number M = 1 was observed. However, at nozzle PR > 1.92, specifically 3 to 5, the nozzle becomes underexpanded with diamond pattern shock wave cells were observed downstream the nozzle lip. The investigated PB and DB solvers show good agreement in terms of flow structures downstream the nozzle exit. Moreover, it was found that as the PR increases, the size of the shock wave cell increases as well, and the streamwise distance of the first shock wave cell moves further downstream the nozzle exit plane. For instance, the distance of the first shock cell is 1.09De, 1.57De, and 2De for PR values of 3, 4, and 5, respectively. The increase in the shock strength was confirmed by a sharp acceleration of the flow downstream the nozzle exit edge. The flow upstream the first shock cell reached a maximum Mach number of M = 3 at PR = 5. The distribution of local flow quantities, such Mach number, and turbulent kinetic energy showed that PB and DB solvers were strongly correlated. However, both solvers overestimated the maximum value of the Mach number upstream the Mach disc with deviation of 4.5%. The distribution of turbulent kinetic energy, showed that the DB solver over predict the turbulence level at a steamwise location X > 10, as the flow becomes subsonic in this region. It can be concluded that although PB solver is normally used to solve low speed incompressible flows, it can be effectively implemented to predict shock structures downstream underexpanded converging nozzles. Further studies should be performed to investigate the effectiveness of this solver in other cases, such high temperature compressible flow.

## Data Availability

All data supporting the findings of this study are available within the paper.
